# Extended Viral Shedding of MERS-CoV Clade B Virus in Llamas Compared with African Clade C Strain

**DOI:** 10.3201/eid2903.220986

**Published:** 2023-03

**Authors:** Jordi Rodon, Anna Z. Mykytyn, Nigeer Te, Nisreen M.A. Okba, Mart M. Lamers, Lola Pailler-García, Guillermo Cantero, Irina Albulescu, Berend-Jan Bosch, Malik Peiris, Albert Bensaid, Júlia Vergara-Alert, Bart L. Haagmans, Joaquim Segalés

**Affiliations:** Unitat Mixta d'Investigació IRTA-UAB en Sanitat Animal, Centre de Recerca en Sanitat Animal (CReSA), Campus de la Universitat Autònoma de Barcelona, Bellaterra, Spain (J. Rodon, N. Te, L. Pailler-García, G. Cantero, A. Bensaid, J. Vergara-Alert, J. Segalés);; IRTA, Programa de Sanitat Animal, CReSA, Campus de la Universitat Autònoma de Barcelona, Bellaterra (J. Rodon, N. Te, L. Pailler-García, G. Cantero, A. Bensaid, J. Vergara-Alert);; Erasmus Medical Centre, Rotterdam, the Netherlands (A.Z. Mykytyn, N.M.A. Okba, M.M. Lamers, B.L. Haagmans);; Utrecht University, Utrecht, the Netherlands (I. Albulescu, B.-J. Bosch);; The University of Hong Kong, Hong Kong, China (M. Peiris);; Facultat de Veterinària, Universitat Autònoma de Barcelona, Bellaterra (J. Segalés)

**Keywords:** MERS-CoV, camelid, llama, Middle East respiratory syndrome coronavirus, MERS-CoV clade C, virus transmission, viruses, Spain

## Abstract

Middle East respiratory syndrome coronavirus (MERS-CoV) clade B viruses are found in camelids and humans in the Middle East, but clade C viruses are not. We provide experimental evidence for extended shedding of MERS-CoV clade B viruses in llamas, which might explain why they outcompete clade C strains in the Arabian Peninsula.

Middle East respiratory syndrome coronavirus (MERS-CoV) infections cause severe pneumonia, acute respiratory distress syndrome, and even lethal disease in humans. High case-fatality rates are reported in the Middle East (*1*), where the virus is endemic and represents a major human health threat. Although major travel-associated outbreaks have occurred and nosocomial transmissions have been documented, MERS-CoV is primarily carried and transmitted to humans by dromedary camels, which are the natural reservoirs and main source of zoonotic events (*2*). All primary cases of MERS-CoV in humans reported during July–December 2021 occurred in persons who had been exposed to dromedary camels (*3*). Susceptible camelid species, such as dromedaries, llamas, and alpacas (*4*), as opposed to humans, do not experience severe disease upon MERS-CoV infection. Infection in camelids is characterized by upper respiratory tract replication, abundant infectious viral shedding, and high transmission potential (*2*). Furthermore, robust and transient innate immune responses in alpacas correlate with virus clearance in the respiratory epithelia (*5*,*6*).

High seroprevalences and active circulation of MERS-CoV have been determined in dromedary camels from the Arabian Peninsula and Africa (*7*). Although >80% of the global camel population is found in Africa (https://www.fao.org/faostat) and MERS-CoV infection is widespread in dromedaries in Africa, zoonotic disease has only been reported in the Arabian Peninsula. Serologic and molecular evidence of MERS-CoV infection in camel handlers exists (*8*–*11*), but no zoonotic transmission has been reported in Africa. Despite continuous trade of dromedaries into the Arabian Peninsula, African clade C MERS-CoV strains have not been detected in the region. One explanation for the dominance of clade B strains in the Middle East could be their increased fitness compared with African clade C viruses. A recent study demonstrated increased replication competence of MERS-CoV clade B Arabian viruses compared with different clade C African strains in human lung ex vivo cultures and in a transgenic mouse model expressing the human cell receptor for MERS-CoV (human dipeptidyl peptidase-4 [hDPP4]) (*12*). However, the replication and transmission competence of Arabian and African viruses in camelid reservoir species remains unknown. 

The kinetics by which llamas shed infectious MERS-CoV are similar to those of dromedary camels, so they are considered a reliable surrogate model for transmission experiments (*2*,*4*). Therefore, we experimentally investigated transmission of MERS-CoV viruses in llamas.

## The Study

We used a previously developed direct-contact model in which the transmission of a MERS-CoV clade B isolate (Qatar15/2015) was assessed in llamas (*13*,*14*). In brief, we kept a group of 5 llamas inside an experimental enclosure (Appendix Figure 1, panel A) to study the transmission capabilities of a MERS-CoV clade C isolate (MERS-CoV/Egypt2013) that was obtained from an infected dromedary (*15*). We inoculated 2 llamas with MERS-CoV and placed them in direct contact with 3 sentinels at 2 days postinoculation (dpi) (Appendix). We monitored clinical signs and body temperature and collected nasal swab specimens for virologic studies. In addition, we retrieved experimental data from MERS-CoV Qatar15/2015-inoculated and in-contact llamas (*13*,*14*) and performed comparative analyses.

We specifically selected the animals used in the transmission studies to be 6-to-10-month-old juveniles of similar geographic origin, sex, and health status background. All animal experimentation and MERS-CoV handling were conducted at the Biosafety Level 3 facilities of the Biocontainment Unit of IRTA CReSA (Barcelona, Spain). Animal handling and experimental procedures were approved by the Ethical and Animal Welfare Committee of IRTA and by the Ethical Commission of Animal Experimentation of the Autonomous Government of Catalonia (approval nos. FUE-2017–00561265 and CEA-OH/10942/1).

**Figure 1:**
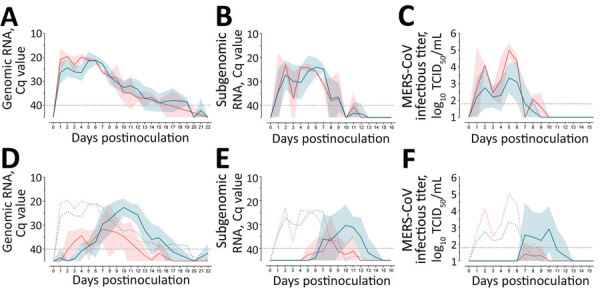
MERS-CoV RNA and infectious virus shedding in llamas experimentally infected with MERS-CoV Egypt/2013 (red) or Qatar15/2015 (blue) strains. A–C) Viral RNA and infectious MERS-CoV shedding of inoculated animals. Genomic (A) and subgenomic (B) viral RNA were quantified in nasal swab samples collected at different times after MERS-CoV inoculation. Infectious MERS-CoV titers (C) were demonstrated in nasal swab specimens collected on different days after MERS-CoV inoculation. Solid lines indicate mean values determined for different MERS-CoV–inoculated groups; shadings represents SD intervals. D–F) Infection profile of naive in-contact llamas). Genomic (D) and subgenomic (E) viral RNA quantified in nasal swab samples collected at different times after MERS-CoV inoculation. Infectious MERS-CoV titers (F) were demonstrated in nasal swab samples collected on different days after MERS-CoV inoculation. Solid lines indicate mean values of the groups of animals infected by contact; shaded areas represent SD intervals. Colored dashed lines indicate mean values calculated for MERS-CoV–inoculated animals. Horizontal dashed lines depict detection limits of assays. Cq, quantification cycle; MERS-CoV, Middle East respiratory syndrome coronavirus; TCID_50_, 50% tissue culture infective dose.

Rectal temperatures of all animals remained at basal levels (37°C–40°C), and no animals displayed clinical signs during the study. We detected no gross or microscopic lesions in the upper or lower respiratory tracts of any studied llama, independent of their experimental group. Animals inoculated with a high dose of either MERS-CoV Egypt/2013 (clade C) or Qatar15/2015 (clade B) had similar levels of genomic and subgenomic viral RNA in nasal swab specimens for 2 weeks (Figure 1, panels A, B). They also shed high titers of infectious virus during the first week after inoculation in a similar biphasic pattern (Figure 1, panel C), indicating that the doses used to inoculate the animals caused productive infection with both strains. Although viral shedding was comparable between both experimental groups, higher infectious titers were detected in Egypt/2013-inoculated llamas. The infection was characterized by a first peak of shedding at 2 dpi and a subsequent reduction in MERS-CoV viral loads, followed by a secondary peak before viral clearance.

**Figure 2:**
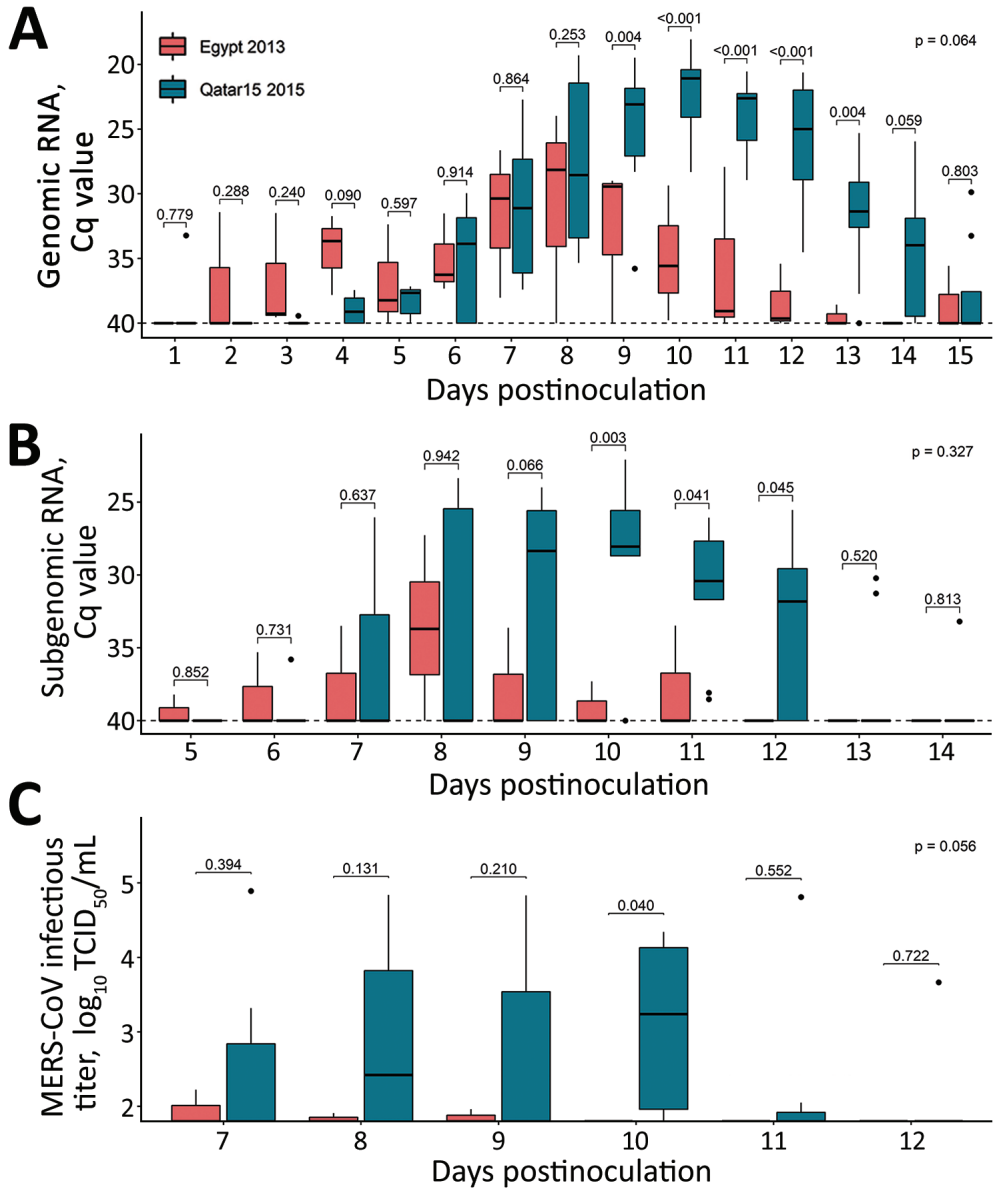
Mixed model analyzing the transmission competence of each MERS-CoV strain over time in investigation of llamas experimentally infected with MERS-CoV. Boxplots show daily virus shedding of sentinel llamas infected with MERS-CoV Egypt/2013 (red) or Qatar15/2015 strains (blue) after direct exposure to inoculated llamas. A, B) Genomic (A) and subgenomic (B) viral RNA quantification in nasal swabs collected throughout the study. C) Infectious MERS-CoV titers. Only the time points considered in the mixed models are represented. Horizontal lines within boxes indicate medians; box tops and bottoms indicate interquartile ranges; error bars indicate 95% CIs; black dots indicate outliers. p values are indicated above the boxes; p values indicate statistical differences between areas under the curve of the experimental groups, as calculated by Wilcoxon test. Cq, quantification cycle; MERS-CoV, Middle East respiratory syndrome coronavirus; TCID_50_, 50% tissue culture infective dose.

The African MERS-CoV isolate was transmitted to 2 of 3 in-contact animals in this study, as determined by quantitative reverse transcription PCR (Figure 1, panels D, E), but infectious virus shedding in contact animals largely remained below threshold levels (Figure 1, panel F). Infectious MERS-CoV Egypt/2013 could only be isolated sporadically and at titers close to the limit of detection. In contrast, the Arabian MERS-CoV Qatar15/2015 isolate was transmitted to all direct-contact llamas, leading to productive infection (Figure 1, panels D–F). Of note, genomic and subgenomic MERS-CoV Egypt/2013 RNA was detected at lower levels and cleared faster in direct-contact llamas than in sentinels infected with the MERS-CoV Qatar15/2015 strain (Figure 1, panels D, E). In the remaining sentinel, a productive infection did not develop, but the animal was naturally exposed to MERS-CoV Egypt/2013, as indicated by traces of genomic RNA in NS at 3–7, 10, and 12 dpi (cycle quantitation values >37) and development of serum neutralizing antibodies (nAbs) to MERS-CoV (Appendix Figure 2). Subgenomic RNA analyses indicated no evidence for either viral replication or shedding in this llama throughout the study. Statistical analyses in sentinel animals showed a significant reduction in MERS-CoV Egypt/2013 replication and shedding period compared with those observed in llamas exposed to MERS-CoV Qatar15/2015 strain (Figure 2). Regardless of the MERS-CoV strain investigated, nAbs were detected starting at 2 weeks after infection in all inoculated animals and in-contact sentinels (Appendix Figure 2). We did not find statistical differences in serum nAb levels among experimental groups. 

Altogether, our data demonstrated transmission of both MERS-CoV strains, which resulted in decreased viral replication and shedding capabilities of the Egypt/2013 strain compared with the Qatar15/2015 strain in sentinel llamas infected by contact. Therefore, the Egypt/2013 strain might have a lower potential of transmission than the Qatar15/2015 strain.

## Conclusions

The results of our experimental investigation might explain why MERS-CoV clade C strains have not been established in the Arabian Peninsula after being introduced through imported camels and competing with enzootic clade B viruses. However, further studies are needed to determine whether this potentially reduced transmissibility is a common feature of the diverse MERS-CoV lineages found in dromedaries in Africa. Specific amino acid substitutions in the spike protein or in other genomic regions of African clade C viruses might be determinant of the low replication phenotype observed in the in-contact animals in our study, as has been previously observed in human cells (*12*). However, viral or host factors that play a key role in conferring replication and transmission competence remain to be explored in camelid reservoirs. High MERS-CoV genome stability was previously described in llamas infected with the Qatar15/2015 strain (*13*). Thus, eventual mutations arising from animals infected with the Egypt/2013 strain were not expected, and no sequencing was performed in those infected animals. Nonetheless, our study provides in vivo experimental data demonstrating reduced MERS-CoV fitness of 1 African clade C isolate to in-contact camelids compared with an Arabian clade B isolate. In addition, reduced MERS-CoV shedding from infected camelids might limit spillover to humans. Introducing MERS-CoV clade B strains to Africa through infected camelids must be avoided, because these strains might outcompete African MERS-CoV clade C strains and pose a greater zoonotic and pandemic threat in Africa.

Appendix Additional information about extended viral shedding in MERS-CoV clade B virus in llamas in comparison with African clade C strain
