# Supplementary material for: Extended Viral Shedding of MERS-CoV Clade B Virus in Llamas Compared with African Clade C Strain
Source: Emerg Infect Dis. 2023 Mar;29(3):585–9. doi: 10.3201/eid2903.220986 (PMC9973703; doi:10.3201/eid2903.220986)
Supplement: Appendix — Additional information about extended viral shedding in MERS-CoV clade B virus in llamas in comparison with African clade C strain [file 22-0986-Techapp-s1.pdf]

Article DOI: https://doi.org/10.3201/eid2903.220986

Extended Viral Shedding of MERS-CoV Clade B Virus in Llamas Compared with African Clade C Strain

Appendix

Materials and Methods

Cell Culture and Viruses

Vero cells (CRL-1586, ATCC, USA) were cultured in Dulbecco's modified Eagle medium (DMEM; Lonza, Switzerland) supplemented with 5% fetal calf serum (EuroClone, Italy), 100 U/mL penicillin, 100 µg/mL streptomycin, and 2 mM glutamine (all ThermoFisher Scientific, USA). Calu-3 cells were cultured in Opti-MEM I (1X) supplemented with GlutaMAX (GIBCO, USA), 10% fetal calf serum, 100 U/mL penicillin and 100 µg/mL streptomycin. MERS-CoV Qatar15/2015 (clade B strain; GenBank accession no. MK280984.2) passage-3 stocks were prepared as previously described (*1*,*2*), and a passage 6 of the MERS-CoV Egypt/2013 strain (*3*) (clade C; GenBank accession no. KJ477103) was propagated for 3 days at 37°C and 5% CO_2 in Vero cells. Viral stocks were sequenced (GenBank accession no. OP906306) to ensure that critical mutations for virion infectivity (in the RBD or the S1/S2 cleavage site) were not acquired in cell culture. Three aa mutations were detected in the Egypt/2013 inoculum: D1418G (ORF1ab), S1251F (Spike, S2 subunit), T8I (M). Infectious virus titers were determined in Vero cells and calculated by determining the dilution that caused 50% cytopathic effect (CPE) in cell cultures (50% tissue culture infectious dose endpoint, $TCID_{50}$).

Study Design

To study the transmission of a MERS-CoV clade C strain, five healthy llamas were purchased from a private animal facility and housed at the animal BSL-3 facilities of the IRTA-CReSA Biocontainment Unit (Barcelona, Spain). The experimental box was set up as in previous MERS-CoV transmission studies (*1*,*2*,*4*) (Appendix Figure 1, panel A). Two llamas were

intranasally inoculated with $10^{6.4}$ $TCID_{50}$ of MERS-CoV Egypt/2013 strain in 3 mL saline solution, using a nebulization device (LMA MADgic, Teleflex Inc., USA) and administrating 1.5 mL into each nostril. At 2 days postinoculation (dpi), inoculated llamas were placed in direct contact with the remaining three sentinel llamas (Appendix Figure 1, panel B). Clinical signs of all animals were monitored for 3 weeks, and rectal temperatures were recorded until 15 dpi with a fast display digital thermometer (AccuVet, Infratec, Italy). Nasal swabs were obtained daily until 15 dpi, plus at 17 and 22 dpi. Whole blood samples of all animals were collected from the jugular vein using Vacutainer tubes (Beckton Dickinson, USA) and serum samples were obtained before MERS-CoV challenge and at 7, 14 and 22 dpi. Animals were euthanized at 22 dpi with an overdose of pentobarbital, followed by a complete necropsy with special focus on upper and lower respiratory tract lesions.

Additionally, aiming to understand differential transmission patterns between MERS-CoV clades, experimental data was retrieved from previous MERS-CoV Qatar15/2015 strain (clade B) transmission studies in llamas and new analyses were performed (*1*,*2*). Briefly, the MERS-CoV Qatar15/2015 strain was previously used to demonstrate transmission from experimentally infected llamas to naïve animals, and to evaluate the efficacy of two distinct vaccine prototypes in blocking virus transmission (*1*,*2*). Animals used in these studies were also allocated in the biocontainment facilities of IRTA-CReSA and experimental procedures followed the same study design, sample collection and the experimental process than the present study with MERS-CoV Egypt/2013 strain. Therefore, data obtained from these studies were considered appropriate for further comparative analyses. Viral RNA and infectious MERS-CoV Qatar15/2015 (clade B) shedding data from eight inoculated and nine contact animals was used to compare the transmissibility versus the Egypt/2013 (clade C) strain in camelids. All non-protected animals met the inclusion criteria and were used for subsequent comparative analyses.

MERS-CoV RNA Detection

Viral RNA was extracted from nasal swab samples with the IndiMag pathogen kit (Indical Biosciences, Germany) using a Biosprint 96 workstation (Qiagen, Germany), following the manufacturer's instructions. Genomic and subgenomic RNA extracts were detected by the UpE and M mRNA RT-qPCR assays, respectively (*5*,*6*). Viral RNA was detected from nasal swabs by RT-qPCR as previously described (*1*,*2*,*4*,*7*–*9*). RT-qPCR was carried out with the AgPath-ID One-Step RT-PCR Reagents (Applied Biosystems, Life Technologies, USA) and

amplification was performed using a 7500 Fast Real-Time PCR System (Applied Biosystems, Life Technologies, USA). Thermal profile followed 10 min at 50°C, 10 sec at 95°C, and 45 cycles of 15 s at 95°C and 30 sec at 58°C. Samples with a quantification cycle (Cq) value ≤40 were considered positive for MERS-CoV genomic or subgenomic RNA.

Virus Titration

The presence of infectious MERS-CoV in nasal swab specimens was evaluated in Vero E6 cells, as previously reported (*1*,*2*,*4*,*7–10*). Briefly, an initial 1/10 dilution and subsequent ten-fold dilutions of the samples were transferred to Vero E6 monolayers. Cells were monitored daily under a light microscope and they were evaluated for the presence of virus-induced cytopathic effect at 6 days after inoculation. The infectious virus concentration in nasal swabs was calculated by determining the dilution that caused 50% CPE in cell cultures ($TCID_{50}$/mL). The limit of detection of the technique was established at 1.8 TCID50/mL.

Plaque Reduction Neutralization Assay

Sera samples collected weekly were tested for the presence of neutralizing antibodies against MERS-CoV (EMC/2012 isolate; GenBank accession no. NC_019843.3) using a plaque reduction neutralization (PRNT) assay according to a previously published protocol (*1*,*2*,*10*), with minor modifications. Briefly, serum samples were inactivated at 56°C for 30 min. Then, 50 µl of 2-fold serially diluted sera were mixed 1:1 with 400 PFU of MERS-CoV, transferred to Calu-3 cells monolayers and incubated at 37°C and 5% CO_2. After 8 h of infection, cells were fixed, permeabilized with 70% ethanol, and stained using mouse anti-MERS-CoV nucleocapsid protein (SinoBiological, China; diluted 1:1000 in 0.1% bovine serum albumin (BSA) -PBS) followed by goat anti-mouse Alexa Fluor 488 antibody (Invitrogen, 1:2000 in 0.1% BSA in PBS). Plates were scanned on the Amersham Typhoon Biomolecular Imager (GE Healthcare, USA). Data was analyzed using ImageQuantTL 8.2 image analysis software (GE Healthcare). The PRNT90 titer was defined as the reciprocal value of the sample dilution that showed 90% reduction of virus growth. Dose-response curves of serum samples were adjusted to a nonlinear fit regression model in Graphpad Prism 9 software, with bottom constraints of 0% and top constraints of 100%. Sera samples from previous studies (*1*,*2*) were re-analyzed to fulfil the above-described criteria.

Data Analyses

To statistically evaluate the transmission potential of each strain, we used two different approaches. First, genomic and subgenomic RNA, and infectious MERS-CoV shedding data were used to calculate areas under the curve of each animal. A Wilcoxon Test was applied to compare the shedding of MERS-CoV/Egypt2013 and MERS-CoV Qatar15/2015 strains within inoculated or in-contact llamas. Second, to compare the results at each time point, a mixed model was adjusted using the shedding data of each individual as a fixed factor and the corresponding MERS-CoV strain and days post-inoculation as random factors, along with a contrast of the estimated marginal means. Only the days whose values differed from the limit of detection were used for the mixed models, which encompassed values from 1 to 15 dpi for genomic RNA, from 5 to 14 dpi for subgenomic RNA and from 7 to 12 dpi for infectious virus. The model validation showed that the residuals of infectious virus shedding data were not normally distributed. Therefore, a log(x+1) transformation was applied only for this dataset. All analyses were performed using *DescTools* (https://cran.r-project.org/web/packages/DescTools/citation.html), *emmeans* (https://github.com/rvlenth/emmeans), *lme4* (https://cran.r-project.org/web/packages/lme4/citation.html), *stats*, and *tidyverse* (https://joss.theoj.org/papers/10.21105/joss.01686) statistic packages for the R software (https://www.r-project.org/). Results of the statistical analyses in sentinel animals are shown in Figure 2.

References

1. Rodon J, Okba NMA, Te N, van Dieren B, Bosch B-J, Bensaid A, et al. Blocking transmission of Middle East respiratory syndrome coronavirus (MERS-CoV) in llamas by vaccination with a recombinant spike protein. Emerg Microbes Infect. 2019;8:1593–603. PubMed https://doi.org/10.1080/22221751.2019.1685912

2. Rodon J, Mykytyn A, Martinez S, Cantero G, Albulescu IC, Bosch B-J, et al. Protective efficacy of an RBD-based Middle East respiratory syndrome coronavirus (MERS-CoV) particle vaccine in llamas. One Health Outlook. 2022;4:12. **PMID 35739576**

3. Chu DKW, Poon LLM, Gomaa MM, Shehata MM, Perera RAPM, Abu Zeid D, et al. MERS coronaviruses in dromedary camels, Egypt. Emerg Infect Dis. 2014;20:1049–53. PubMed https://doi.org/10.3201/eid2006.140299

4. Vergara-Alert J, Raj VS, Muñoz M, Abad FX, Cordón I, Haagmans BL, et al. Middle East respiratory syndrome coronavirus experimental transmission using a pig model. Transbound Emerg Dis. 2017;64:1342–5. PubMed https://doi.org/10.1111/tbed.12668

5. Corman VM, Eckerle I, Bleicker T, Zaki A, Landt O, Eschbach-Bludau M, et al. Detection of a novel human coronavirus by real-time reverse-transcription polymerase chain reaction. Euro Surveill. 2012;17:20285. PubMed https://doi.org/10.2807/ese.17.39.20285-en

6. Coleman CM, Frieman MB. Growth and Quantification of MERS-CoV Infection. Curr Protoc Microbiol. 2015;37:15E.2.1–9. **PMID 26344219**

7. Vergara-Alert J, van den Brand JMA, Widagdo W, Muñoz M V, Raj S, Schipper D, et al. Livestock susceptibility to infection with Middle East respiratory syndrome coronavirus. Emerg Infect Dis. 2017;23:232–40. PubMed https://doi.org/10.3201/eid2302.161239

8. Te N, Rodon J, Ballester M, Pérez M, Pailler-García L, Segalés J, et al. Type I and III IFNs produced by the nasal epithelia and dimmed inflammation are features of alpacas resolving MERS-CoV infection. PLoS Pathog. 2021;17:e1009229. PubMed https://doi.org/10.1371/journal.ppat.1009229

9. Te N, Rodon J, Pérez M, Segalés J, Vergara-Alert J, Bensaid A. Enhanced replication fitness of MERS-CoV clade B over clade A strains in camelids explains the dominance of clade B strains in the Arabian Peninsula. Emerg Microbes Infect. 2022;11:260–74. PubMed

10. Haagmans BL, van den Brand JMA, Raj VS, Volz A, Wohlsein P, Smits SL, et al. An orthopoxvirus-based vaccine reduces virus excretion after MERS-CoV infection in dromedary camels. Science. 2016;351:77–81. PubMed https://doi.org/10.1126/science.aad1283

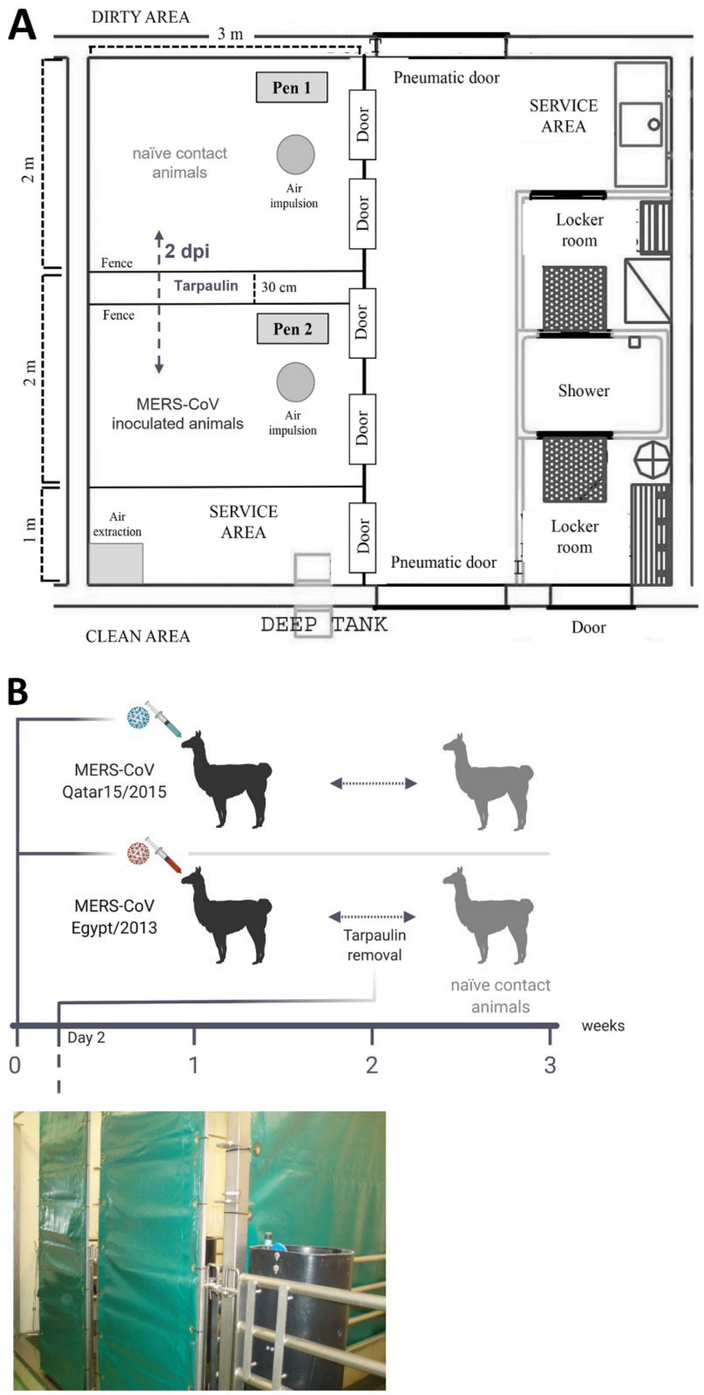

Appendix Figure 1. Schematic representation of the experimental set up. A) A group of llamas (black) was intranasally inoculated with MERS-CoV Qatar15/2015 (blue) or Egypt/2013 (red) and placed in separated experimental boxes. B) Inoculated llamas were kept separated from naïve llamas (gray) using a tarpaulin to avoid direct contact. Two days post MERS-CoV inoculation, tarpaulin was removed, and animals got in direct contact. Afterwards, the viral shedding and humoral responses were monitored for 3 weeks as indicated in the timeline.

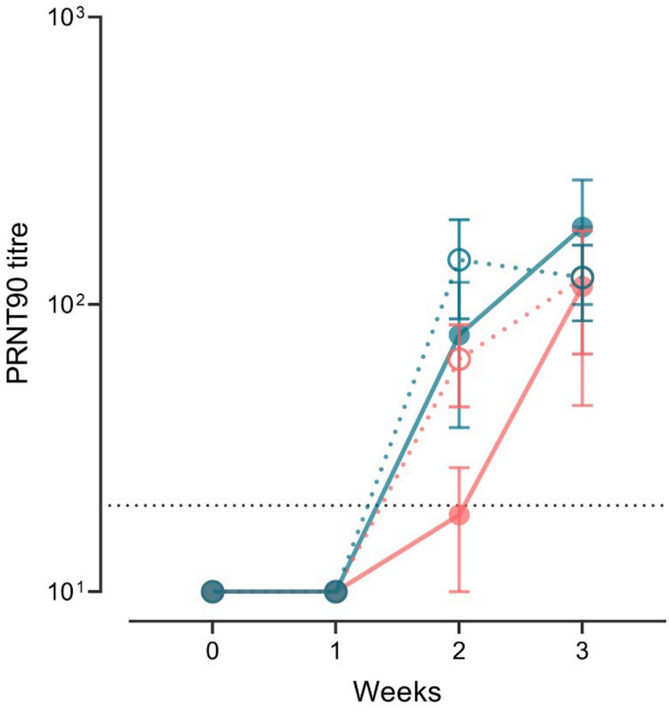

Appendix Figure 2. Development of neutralizing humoral responses by llamas infected with MERS-CoV Egypt/2013 or Qatar15/2015. The plot displays levels of serum neutralizing antibodies elicited in llamas upon MERS-CoV Egypt/2013 (red) or Qatar15/2015 (blue) inoculation (dashed lines) or direct exposition to inoculated ones (solid lines). Each line represents mean group values and error bars represent standard error mean intervals. Grey dashed lines depict the detection limits of the assays. PRNT90, 90% plaque reduction neutralization titer.
